# Gender differences in home care clients and admission to long-term care in Ontario, Canada: a population-based retrospective cohort study

**DOI:** 10.1186/1471-2318-13-48

**Published:** 2013-05-16

**Authors:** Andrea Gruneir, Jacqueline Forrester, Ximena Camacho, Sudeep S Gill, Susan E Bronskill

**Affiliations:** 1 Women’s College Research Institute, Women’s College Hospital, Toronto, Ontario, Canada; 2 Institute for Clinical Evaluative Sciences, Toronto, Ontario, Canada; 3 Institute of Health Policy, Management and Evaluation, University of Toronto, Toronto, Ontario, Canada; 4 Department of Community Health Sciences, University of Calgary, Calgary, Alberta, Canada; 5 Departments of Medicine and Community Health and Epidemiology, Queen’s University, Kingston, Ontario, Canada

**Keywords:** Older women, Older men, Home care, Transitions, Informal care, Long-term care placement, Nursing homes

## Abstract

**Background:**

Home care is integral to enabling older adults to delay or avoid long-term care (LTC) admission. To date, there is little population-based data about gender differences in home care users and their subsequent outcomes. Our objectives were to quantify differences between women and men who used home care in Ontario, Canada and to determine if there were subsequent differences in LTC admission.

**Methods:**

This is a population-based retrospective cohort study. We identified all adults aged 76+ years living in Ontario and receiving home care on April 1, 2007 (baseline). Using the Resident Assessment Instrument – Home Care (RAI-HC) linked to other databases, we characterized the cohort by living condition, health and functioning, and identified all acute care and LTC use in the year following baseline.

**Results:**

The cohort consisted of 51,201 women and 20,102 men. Women were older, more likely to live alone, and more likely to rely on a child or child-in-law for caregiver support. Men most frequently identified a spouse as caregiver and their caregivers reported distress twice as often as women’s caregivers. Men had higher rates of most chronic conditions and were more likely to experience impairment. Men were more likely to be admitted to hospital, to have longer stays in hospital, and to be admitted to LTC.

**Conclusions:**

Understanding who uses home care and why is critical to ensuring that these programs effectively reduce LTC use. We found that women outnumbered men but that men presented with higher levels of need. This detailed gender analysis highlights how needs differ between older women, men, and their respective caregivers.

## Background

Aging brings different health and social challenges for women and men. In developed nations, women tend to live longer but experience more chronic illness, disability, and health care use than men [1-3]. While men have been described as having a higher burden of “mortal conditions” including heart disease and diabetes, women have been described as experiencing more “morbid conditions” such as arthritis and falls [2]. The implications of these health-related differences are compounded by older women’s and men’s different social conditions [4,5]. Older women are more likely to rely on non-spousal caregivers for support, often children, due to higher rates of widowhood and lower rates of re-marriage. Even within older couples, women are more likely than men to take on the caregiver role since they are often relatively younger and since it is more consistent with historical gender roles [4,5]. Some research suggests that not only do women have less informal support but that they also must exhibit greater levels of disability before it is provided than their male counterparts [5].

For many older adults, formal home care services are an important component in helping them safely remain in the community. Home care has been shown to promote independence [6], reduce future health services utilization [7], and shorten lengths of stay in acute care [8]. Home care has also been shown to reduce caregiver stress when adequate assistance, including respite and emotional support, is provided [9]. Most importantly, home care has been shown to delay admission to long-term care (LTC) facilities [7,10]. From a health system perspective, home care is generally considered less costly than institution-based care [10], while from the recipients’ perspective, it enables them to stay within their own communities for as long as possible. In 2002, The Commission on the Future of Health Care in Canada called for home care to become “the next essential service” in Canadian health care [11].

To ensure that home care meets the needs of older adults and enables them to delay or avoid LTC admission, a clear understanding of who is served by home care is required. While others have described home care users and pertinent outcomes [12-15], few have looked at gender differences. Given the significant differences between older women and men in their health and social profiles in general, it is important to understand if those same differences would be observed among older home care users and how these might affect their subsequent use of acute care and LTC. In this study, our objectives were to use population-based data to quantify differences between older women and men who used long-stay (non-post-acute) home care services in Ontario and to determine whether there were subsequent differences in use of LTC. The rationale behind this study was to gain a better understanding of gender differences in home care use at a population-level in order to inform home care policy and planning.

## Methods

### Setting

This study was conducted using data from Ontario, the largest Canadian province. Ontario is home to approximately 13 million people, the vast majority of whom are covered through a universal, publically-funded health insurance program that includes physician services, inpatient care, home care, and LTC; prescription medications are covered for individuals 65 years and older [16]. The province is divided into 14 health regions, each of which has a Community Care Access Centre (CCAC) that coordinates delivery of provincially-funded community-based services [17] and facilitates the LTC admission process. Home care is provided on either a short- or long-stay basis, where the latter refers to patients who receive care for a minimum of 60 days in a single episode [18]. The specific services and the intensity of services provided vary depending on client need but may consist of homemaking, personal support (i.e. assistance with bathing), and health professional visits. Since the system is publicly funded, service maximums have been put in place; for example, homemaking services cannot exceed 60 hours in a 30-day period [19]. LTC homes, also known as nursing homes, provide care to adults who require 24-hour nursing services and/or supervision.

### Data sources

We used multiple encrypted and linked population-based administrative databases including: the Registered Persons Database (RPDB), which includes demographic information on all Ontario residents; the Resident Assessment Instrument for Home Care (RAI-HC), a comprehensive clinical assessment tool mandated for use on all long-stay home care clients in Ontario [20-22]; the Canadian Institute for Health Information Discharge Abstract Database (DAD), which consists of standardized chart abstractions for all inpatient hospital episodes; the National Ambulatory Care Reporting System (NACRS), which consists of standardized reporting on all emergency department (ED) visits; the Ontario Health Insurance Plan (OHIP) billing claims database for physician visits; the Ontario Drug Benefits (ODB) claims database for outpatient prescriptions; and the Client Profile Database which centralizes information on all applications and admissions to LTC. These data are regularly used for research purposes [23-25] and have been studied extensively for validity. These data are housed at the Institute for Clinical Evaluative Sciences (ICES) which is a prescribed entity under Ontario Privacy legislation and is permitted to hold and use the described data files for research and evaluation purposes under strict privacy procedures. The data were linked at the individual level using unique encrypted identifiers. This study was approved by the research ethics board of Sunnybrook Health Sciences Centre.

### Cohort

We identified all Ontario residents aged 76 years or older who were receiving home care services as of April 1, 2007 (baseline); we restricted inclusion to long-stay recipients by identifying those with a completed RAI-HC assessment in the year prior to baseline. We chose 76 years as the lower age limit to focus on adults most likely to be at risk of LTC admission. For each cohort member, we used a one-year look-back window to capture health services use in the administrative claims data for the full year prior to cohort entry. When there was more than one RAI-HC assessment, we chose the assessment closest to baseline. We further stratified our cohort by gender and five-year age groups.

### Sociodemographic measures

Data on living situation, presence of and relationship to a primary caregiver, and caregiver distress were obtained from the RAI-HC. We used two measures of socioeconomic status. The first measure stratified individuals into quintiles based on mean neighbourhood income that was derived from Statistics Canada’s national census. The second was based on an individual’s qualification for prescription drug co-payment subsidies [26]. Older adults with an annual income below a specified threshold qualify for a reduction in their required co-payment for outpatient prescription medications; this is identified by a special flag on the ODB claim.

### Health status measures

We measured health status using several of the RAI-HC embedded outcome scales. The Cognitive Performance Scale (CPS) uses items on memory and communication skills to create a 7-point scale; scores ≥3 are indicative of moderate to severe cognitive impairment [27]. The Activities of Daily Living (ADL) Hierarchy Scale uses information on self-performance of ADLs, such as bathing and eating, to assign scores from 0 (no impairment) to 6 (total dependence on care); for this study, ADL impairment was defined as a score ≥1 [23,28]. The Instrumental Activities of Daily Living (IADL) Difficulty Scale measures difficulties with daily tasks, such as light housework and banking, to assign scores from 0 to 6 [29]; IADL impairment was defined as a score ≥4 [30]. The Changes in Health, End-stage Disease, and Signs and Symptoms (CHESS) Scale identifies individuals at risk for significant decline. It consists of six items to create a scale with scores ranging from 0 to 5 [31], with a score of ≥2 indicating instability. The Depression Rating Scale (DRS) consists of seven items to create a 14-point scale where scores ≥3 serve as a marker for depression [32]. The Method for Assigning Priority Levels (MAPLe) differentiates clients into five priority levels based on their risk of health decline. It is derived from measures of cognitive impairment, ADL difficulty, problem behaviours, and identified risk for long-term care admission [22]. We present scale score cut-offs, rather than the full scale distribution, to be consistent with other presentations of RAI outcome scores [33,34].

We used physician claims (OHIP) and hospital data (DAD and NACRS) to identify International Classification of Disease versions 9 and 10 (ICD-9/10) codes for medical illnesses and fractures recorded in the 5 years prior to baseline. A full list of diagnoses and corresponding ICD-9/10 codes can be found in Additional file 1. We summed the number of medical illnesses to create a measure of multi-morbidity, as has been done elsewhere [35]. The total number of unique prescription medications used in the year prior to baseline [36] served as a secondary measure of multi-morbidity.

### Health service use measures

We examined health services use in the year following baseline.

We identified all-cause emergency department (ED) visits as well as potentially preventable visits and visits for fall-related injuries. Potentially preventable ED visits, sometimes referred to as visits for ambulatory care sensitive conditions, are visits for conditions which are generally amenable to primary care for the prevention of acute complications. Examples of such visits include exacerbations of chronic conditions (i.e. chronic obstructive pulmonary disease, diabetes, or congestive heart failure) and complications of common infections (i.e. dehydration and pneumonia). The concept of potentially preventable conditions is regularly used to study access to primary care [37-40].

For patients admitted to hospital, we measured the total length of stay, designation as “alternate level of care” (ALC), and length of stay in ALC. ALC designations are given to patients who no longer require hospital-level services but cannot be discharged because appropriate care is not available elsewhere (for example, when a LTC bed is not available for a patient who requires one). ALC patients have been identified as a major contributor to hospital bed shortages and ED backlogs [34,41].

Finally, we identified all applications and admissions to LTC facilities in the year following baseline. As the length of time between application and admission to LTC can be sizeable, particularly for those applying from the community [42], it is important to capture both dates when considering the role of community-based services in preventing or delaying LTC admission.

### Analyses

We used descriptive statistics to compare older women and men in Ontario’s home care system. Ninety-five percent confidence intervals (95% CIs) were estimated to facilitate comparisons. The decision was made not to use statistical tests given the intent of the study and the large cohort size which would have resulted in small p-values. Analyses were additionally stratified by age to display differential age effects by gender.

## Results

**Figure 1:**
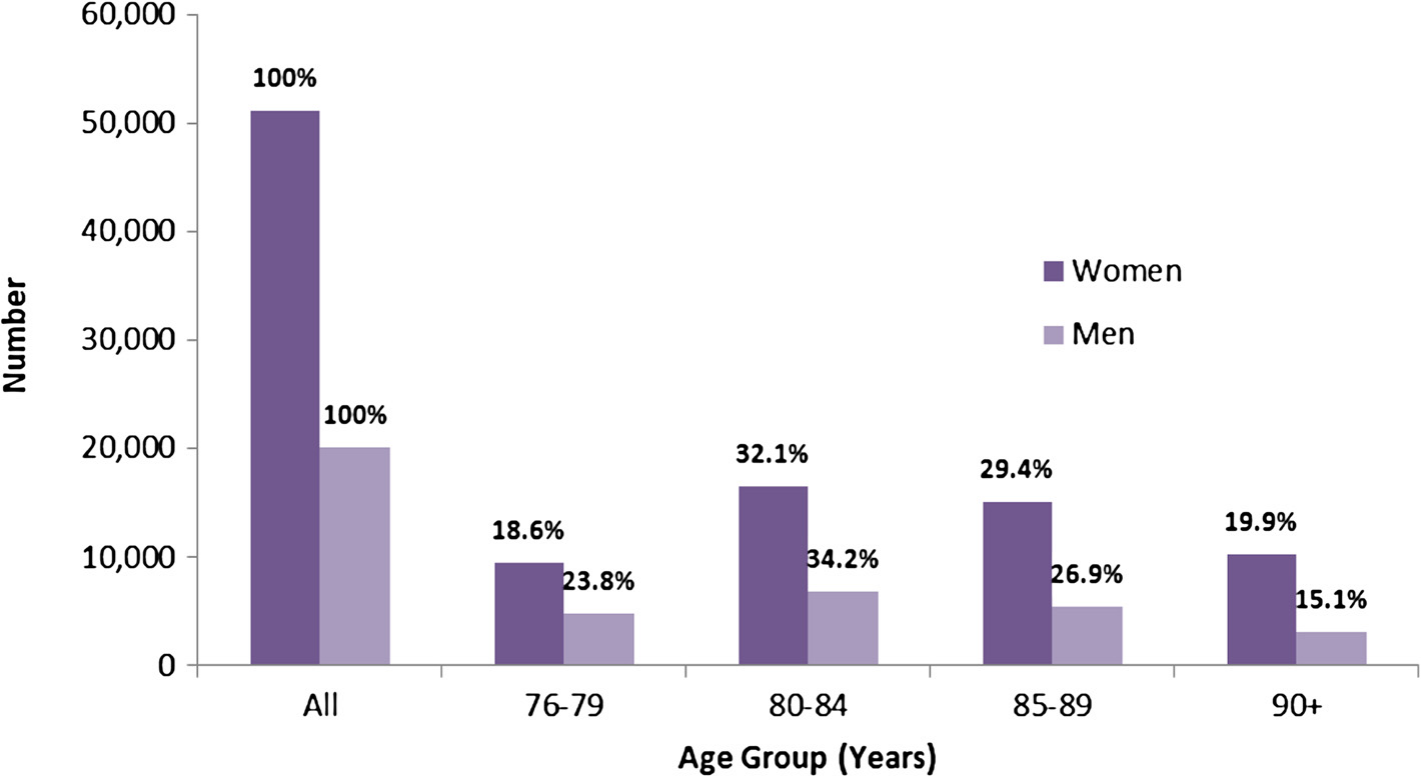
Age and gender distribution of older long-stay home care clients in Ontario, Canada, 2007.

We identified 51,201 women and 20,102 men aged 76 years or older with a completed RAI-HC assessment in Ontario in the year prior to baseline. Women outnumbered men in every age group (Figure 1). The largest proportion of women and men were aged 80–84 years of age.

Overall, women had lower socioeconomic standing than men. In all age groups, there were larger proportions of women in the lowest income quintile and who qualified for prescription co-payment subsidies. Women were more likely to live alone compared to men (15.5% versus 10.5%). Regardless of living situation, women were most likely to report a child or child-in-law as the primary caregiver whereas men were most likely to report a spouse, except among the oldest age group where men also reported a child or child-in-law as the most common primary caregiver. Among those who lived with the primary caregiver, men were still more likely to report that a spouse took on the role of caregiver than were women (76.3% versus 36.6%). Caregiver distress was more commonly observed among caregivers of men than women (18.2% versus 9.9%) (Table 1).

**Table 1:** Sociodemographic and clinical characteristics of older women and men in long-stay home care in Ontario, Canada, 2007 – overall and by age

	**Women N = 51,201**					**Men N = 20,102**				
	**All women**	**Aged 76-79**	**Aged 80-84**	**Aged 85-89**	**Aged 90+**	**All men**	**Aged 76-79**	**Aged 80-84**	**Aged 85-89**	**Aged 90+**
	**N = 51,201**	**N = 9,523**	**N = 16,447**	**N = 15,036**	**N = 10,195**	**N = 20,102**	**N = 4,781**	**N = 6,880**	**N = 5,413**	**N = 3,028**
**Demographic measures, % (95% CI)**										
***Income quintile based on mean neighbourhood income***										
Q1 (Lowest)	25.3%	26.5%	25.7%	25.2%	23.7%	22.8%	23.6%	22.4%	23.8%	20.7%
	(24.9-25.8)	(25.5-27.5)	(25.0-26.5)	(24.4-26.0)	(22.8-24.7)	(22.1-23.5)	(22.2-25.0)	(21.3-23.6)	(22.5-25.1)	(19.1-22.4)
Q2	21.5%	22.3%	21.7%	21.6%	20.5%	21.5%	21.8%	21.9%	20.9%	21.5%
	(21.1-22.0)	(21.4-23.3)	(21.0-22.4)	(20.9-22.4)	(19.6-21.4)	(20.9-22.2)	(20.5-23.1)	(20.8-23.0)	(19.7-22.1)	(19.9-23.2)
Q3	18.8%	18.6%	18.8%	18.6%	19.1%	19.2%	19.7%	19.2%	18.8%	19.3%
	(18.4-19.2)	(17.7-19.5)	(18.2-19.5)	(17.9-19.3)	(18.3-20.0)	(18.6-19.8)	(18.4-21.0)	(18.2-20.3)	(17.7-20.0)	(17.8-21.0)
Q4	17.7%	17.7%	17.6%	17.9%	17.5%	18.6%	18.4%	19.5%	18.3%	17.6%
	(17.3-18.1)	(16.8-18.5)	(17.0-18.3)	(17.3-18.6)	(16.7-18.3)	(18.0-19.2)	(17.2-19.6)	(18.5-20.6)	(17.2-19.5)	(16.1-19.1)
Q5 (Highest)	16.4%	14.8%	15.9%	16.4%	18.7%	17.5%	16.3%	16.8%	17.9%	20.5%
	(16.0-16.7)	(14.0-15.6)	(15.3-16.5)	(15.8-17.1)	(17.9-19.6)	(17.0-18.1)	(15.2-17.5)	(15.9-17.8)	(16.8-19.1)	(18.9-22.2)
Prescription co-payment subsidy	41.9%	40.0%	39.7%	41.5%	47.8%	26.2%	25.5%	25.0%	25.7%	31.0%
	(41.3-42.5)	(38.7-41.3)	(38.8-40.7)	(40.5-42.5)	(46.4-49.1)	(25.5-26.9)	(24.1-27.0)	(23.8-26.2)	(24.4-27.1)	(29.1-33.1)
Living status & caregiver relationship*										
Lived alone	15.5%	16.4%	16.3%	16.2%	12.4%	10.5%	10.2%	11.2%	10.3%	9.8%
	(15.2-15.8)	(15.6-17.3)	(15.7-16.9)	(15.6-16.9)	(11.7-13.1)	(10.1-11.0)	(9.3-11.2)	(10.4-12.0)	(9.4-11.2)	(8.7-11.0)
Co-resided with primary care giver	38.2%	46.6%	40.2%	34.4%	32.7%	62.5%	70.1%	64.4%	58.8%	52.3%
	(37.7-38.8)	(45.3-48.0)	(39.2-41.2)	(33.5-35.4)	(31.6-33.9)	(61.4-63.6)	(67.8-72.6)	(62.6-66.4)	(56.8-60.9)	(49.8-55.0)
***Relationship with primary caregiver (among those who resided together)***										
Chi ld/chi ld-in-law	55.8%	40.4%	50.6%	62.2%	76.7%	19.9%	13.9%	17.2%	22.4%	35.1%
	(54.8-56.9)	(38.6-42.3)	(48.9-52.4)	(60.0-64.4)	(73.7-79.7)	(19.1-20.7)	(12.7-15.3)	(16.0-18.5)	(20.8-24.1)	(32.2-38.1)
Spouse	36.6%	53.8%	43.1%	29.7%	11.4%	76.3%	82.5%	79.0%	74.2%	60.2%
	(35.7-37.4)	(51.7-56.0)	(41.6-44.8)	(28.2-31.2)	(10.2-12.6)	(74.8-77.9)	(79.5-85.6)	(76.4-81.6)	(71.3-77.3)	(56.4-64.1)
Other relative	5.4%	4.1%	4.2%	5.7%	9.0%	2.1%	1.9%	2.1%	1.8%	3.2%
	(5.1-5.7)	(3.6-4.8)	(3.7-4.7)	(5.1-6.4)	(8.0-10.1)	(1.9-2.4)	(1.4-2.4)	(1.7-2.6)	(1.4-2.4)	(2.4-4.2)
Friend/neighbour	2.2%	1.6%	2.0%	2.5%	3.0%	1.7%	1.7%	1.7%	1.6%	1.5%
	(2.0-2.5)	(1.3-2.1)	(1.7-2.4)	(2.1-3.0)	(2.4-3.6)	(1.4-1.9)	(1.3-2.2)	(1.3-2.1)	(1.2-2.1)	(1.0-2.3)
***Relationship with primary caregiver (among all)***										
Child/child-in-law	67.3%	58.3%	65.6%	71.0%	73.0%	38.0%	27.3%	34.6%	42.7%	54.1%
	(66.6-68.0)	(56.8-59.9)	(64.3-66.8)	(69.7-72.4)	(71.4-74.7)	(37.1-38.8)	(25.8-28.8)	(33.2-36.0)	(40.9-44.4)	(51.5-56.8)
Spouse	14.4%	25.6%	17.8%	10.6%	4.0%	48.6%	58.9%	51.7%	44.7%	32.2%
	(14.1-14.7)	(24.6-26.7)	(17.2-18.5)	(10.1-11.1)	(3.6-4.4)	(47.6-49.6)	(56.8-61.1)	(50.0-53.4)	(42.9-46.5)	(30.2-34.3)
Other relative	9.6%	7.6%	8.2%	9.7%	13.7%	5.9%	5.5%	5.8%	5.8%	6.7%
	(9.4-9.9)	(7.0-8.1)	(7.8-8.7)	(9.2-10.2)	(13.0-14.4)	(5.5-6.2)	(4.9-6.2)	(5.2-6.4)	(5.2-6.5)	(5.8-7.7)
Friend/neighbour	7.1%	6.5%	6.8%	7.3%	8.0%	5.9%	6.2%	6.2%	5.4%	5.7%
	(6.9-7.3)	(6.0-7.0)	(6.4-7.2)	(6.8-7.7)	(7.5-8.6)	(5.6-6.2)	(5.5-6.9)	(5.6-6.8)	(4.8-6.0)	(4.9-6.6)
Caregiver experienced distress	9.9%	10.4%	10.4%	9.3%	9.5%	18.2%	18.0%	19.4%	17.4%	17.2%
	(9.6-10.2)	(9.8-11.1)	(9.9-10.9)	(8.8-9.8)	(8.9-10.1)	(17.6-18.8)	(16.9-19.3)	(18.4-20.5)	(16.3-18.6)	(15.7-18.7)
**Diagnosis (Prior 5 years)†**										
Arthritis	74.5%	75.5%	75.9%	75.3%	70.2%	65.5%	64.6%	65.8%	66.0%	65.4%
	(73.8-75.3)	(73.8-77.3)	(74.5-77.2)	(73.9-76.7)	(68.5-71.8)	(64.4-66.6)	(62.3-66.9)	(63.9-67.7)	(63.8-68.2)	(62.5-68.3)
COPD	26.0%	28.1%	26.1%	25.5%	24.6%	36.6%	36.7%	36.7%	36.6%	36.3%
	(25.5-26.4)	(27.1-29.2)	(25.3-26.8)	(24.7-26.3)	(23.6-25.5)	(35.8-37.5)	(35.0-38.5)	(35.3-38.2)	(35.0-38.3)	(34.2-38.5)
Cancer	7.3%	9.8%	7.9%	6.5%	5.3%	13.9%	16.6%	14.3%	13.3%	9.8%
	(7.1-7.6)	(9.1-10.4)	(7.5-8.4)	(6.1-6.9)	(4.9-5.8)	(13.4-14.4)	(15.5-17.8)	(13.4-15.2)	(12.3-14.3)	(8.8-11.0)
Diabetes	28.4%	37.1%	31.6%	25.6%	19.5%	35.8%	42.7%	38.3%	33.1%	24.3%
	(28.0-28.9)	(35.9-38.4)	(30.7-32.5)	(24.7-26.4)	(18.6-20.4)	(35.0-36.7)	(40.9-44.6)	(36.8-39.8)	(31.6-34.7)	(22.6-26.1)
Cardiovascular disease	19.8%	19.6%	19.7%	19.8%	20.0%	27.3%	28.2%	28.0%	26.4%	25.9%
	(19.4-20.2)	(18.8-20.6)	(19.1-20.4)	(19.0-20.5)	(19.2-20.9)	(26.6-28.0)	(26.7-29.7)	(26.7-29.2)	(25.0-27.8)	(24.1-27.8)
Dementia	31.6%	27.2%	30.9%	33.3%	34.2%	37.6%	33.8%	38.1%	40.0%	38.3%
	(31.1-32.1)	(26.2-28.3)	(30.1-31.8)	(32.4-34.2)	(33.0-35.3)	(36.8-38.5)	(32.2-35.5)	(36.7-39.6)	(38.3-41.7)	(36.1-40.5)
Osteoporosis	26.4%	26.3%	27.3%	26.9%	24.2%	8.7%	7.7%	9.0%	8.9%	9.3%
	(25.9-26.8)	(25.3-27.3)	(26.5-28.1)	(26.1-27.8)	(23.3-25.2)	(8.3-9.1)	(6.9-8.5)	(8.3-9.7)	(8.1-9.7)	(8.3-10.5)
**Fractures (Prior 5 years)†**										
Wrist/forearm	5.7%	5.3%	6.0%	5.7%	5.6%	2.0%	2.1%	2.3%	1.6%	2.1%
	(5.5-5.9)	(4.8-5.7)	(5.7-6.4)	(5.3-6.1)	(5.1-6.0)	(1.8-2.2)	(1.7-2.6)	(1.9-2.7)	(1.3-2.0)	(1.6-2.6)
Shoulder/upper arm	3.7%	3.9%	3.7%	3.6%	3.5%	1.7%	1.3%	1.8%	1.9%	1.5%
	(3.5-3.8)	(3.5-4.3)	(3.4-4.0)	(3.3-3.9)	(3.2-3.9)	(1.5-1.9)	(1.0-1.7)	(1.5-2.2)	(1.6-2.4)	(1.1-2.0)
Thoracic spine	1.1%	1.0%	1.1%	1.1%	1.2%	0.5%	0.5%	0.6%	0.5%	0.5%
	(1.0-1.2)	(0.8-1.2)	(1.0-1.3)	(1.0-1.3)	(1.0-1.4)	(0.4-0.6)	(0.3-0.8)	(0.4-0.8)	(0.4-0.8)	(0.3-0.8)
Lumbar spine and pelvis	3.9%	2.9%	3.5%	4.3%	4.7%	2.3%	1.9%	2.1%	2.3%	3.1%
	(3.7-4.0)	(2.6-3.3)	(3.2-3.8)	(4.0-4.7)	(4.2-5.1)	(2.1-2.5)	(1.5-2.3)	(1.8-2.5)	(1.9-2.7)	(2.5-3.8)
Hip/femur	9.3%	6.7%	8.1%	10.4%	11.8%	5.1%	4.4%	5.0%	5.0%	6.9%
	(9.0-9.5)	(6.2-7.2)	(7.6-8.5)	(9.9-11.0)	(11.2-12.5)	(4.8-5.5)	(3.8-5.0)	(4.5-5.5)	(4.5-5.7)	(6.0-7.9)
Lower leg/ankle	2.5%	3.0%	2.6%	2.3%	2.2%	1.6%	2.1%	1.6%	1.4%	1.1%
	(2.4-2.7)	(2.7-3.4)	(2.4-2.9)	(2.1-2.6)	(1.9-2.5)	(1.4-1.8)	(1.7-2.5)	(1.3-1.9)	(1.1-1.7)	(0.8-1.6)
**Number of chronic conditions (Prior year)**										
0	0.7%	0.4%	0.6%	0.6%	1.0%	1.1%	1.3%	0.9%	1.2%	1.0%
	(0.6-0.7)	(0.3-0.6)	(0.5-0.7)	(0.5-0.8)	(0.8-1.2)	(0.9-1.2)	(1.0-1.6)	(0.7-1.2)	(0.9-1.5)	(0.7-1.5)
1	7.1%	6.9%	6.7%	7.0%	8.3%	7.9%	7.2%	7.2%	7.9%	10.4%
	(6.9-7.4)	(6.4-7.4)	(6.3-7.1)	(6.6-7.5)	(7.7-8.8)	(7.5-8.3)	(6.5-8.0)	(6.5-7.8)	(7.2-8.7)	(9.3-11.6)
2	19.6%	18.7%	19.0%	19.8%	21.3%	19.4%	19.2%	17.9%	19.9%	21.9%
	(19.2-20.0)	(17.8-19.6)	(18.3-19.7)	(19.0-20.5)	(20.5-22.3)	(18.8-20.0)	(18.0-20.5)	(16.9-18.9)	(18.7-21.1)	(20.3-23.7)
3	25.7%	25.5%	25.6%	25.8%	25.8%	25.9%	25.5%	25.9%	25.4%	27.1%
	(25.2-26.1)	(24.5-26.5)	(24.8-26.4)	(25.0-26.6)	(24.8-26.8)	(25.2-26.6)	(24.1-27.0)	(24.7-27.2)	(24.1-26.8)	(25.3-29.0)
4+	46.9%	48.5%	48.1%	46.9%	43.7%	45.9%	46.8%	48.2%	45.6%	39.6%
	(46.3-47.5)	(47.1-49.9)	(47.1-49.2)	(45.8-48.0)	(42.4-45.0)	(44.9-46.8)	(44.9-48.8)	(46.5-49.8)	(43.8-47.5)	(37.4-41.9)
Unique drugs (Prior year)**										
1–5	14.0%	10.8%	12.2%	14.3%	19.5%	13.5%	11.6%	12.0%	13.5%	20.3%
	(13.7-14.4)	(10.2-11.5)	(11.7-12.8)	(13.7-14.9)	(18.7-20.4)	(13.0-14.1)	(10.6-12.6)	(11.2-12.8)	(12.6-14.5)	(18.7-22.0)
6–9	26.0%	22.1%	24.5%	27.3%	30.4%	25.2%	22.4%	24.6%	26.3%	28.8%
	(25.6-26.5)	(21.2-23.1)	(23.7-25.2)	(26.4-28.1)	(29.3-31.5)	(24.5-25.9)	(21.1-23.8)	(23.4-25.8)	(25.0-27.7)	(27.0-30.8)
10–19	49.0%	51.1%	51.1%	49.1%	43.3%	49.0%	50.1%	50.4%	50.0%	42.5%
	(48.4-49.6)	(49.7-52.6)	(50.1-52.2)	(48.0-50.2)	(42.0-44.6)	(48.1-50.0)	(48.1-52.2)	(48.8-52.1)	(48.1-51.9)	(40.2-44.9)
≥20	10.2%	15.4%	11.5%	8.5%	5.5%	11.0%	14.8%	12.0%	8.9%	6.5%
	(9.9-10.5)	(14.6-16.2)	(11.0-12.1)	(8.1-9.0)	(5.1-6.0)	(10.6-11.5)	(13.7-15.9)	(11.2-12.9)	(8.1-9.8)	(5.7-7.5)

Men had a higher prevalence of most medical conditions including chronic obstructive pulmonary disease (COPD) (36.6% versus 26.0%), cancer (13.9% versus 7.3%), diabetes (35.8% versus 28.4%), cardiovascular disease (27.3% versus 19.8%) and dementia (37.6% versus 31.6%), but women were more prone to diagnoses such as osteoporosis (26.4% versus 8.7%) and arthritis (74.5% versus 65.5%). Women were more likely to have experienced all types of fractures than men at every age group. Women and men, however, appeared similar in their cumulative burden of multi-morbidity and drug use (Table 1).

**Figure 2:**
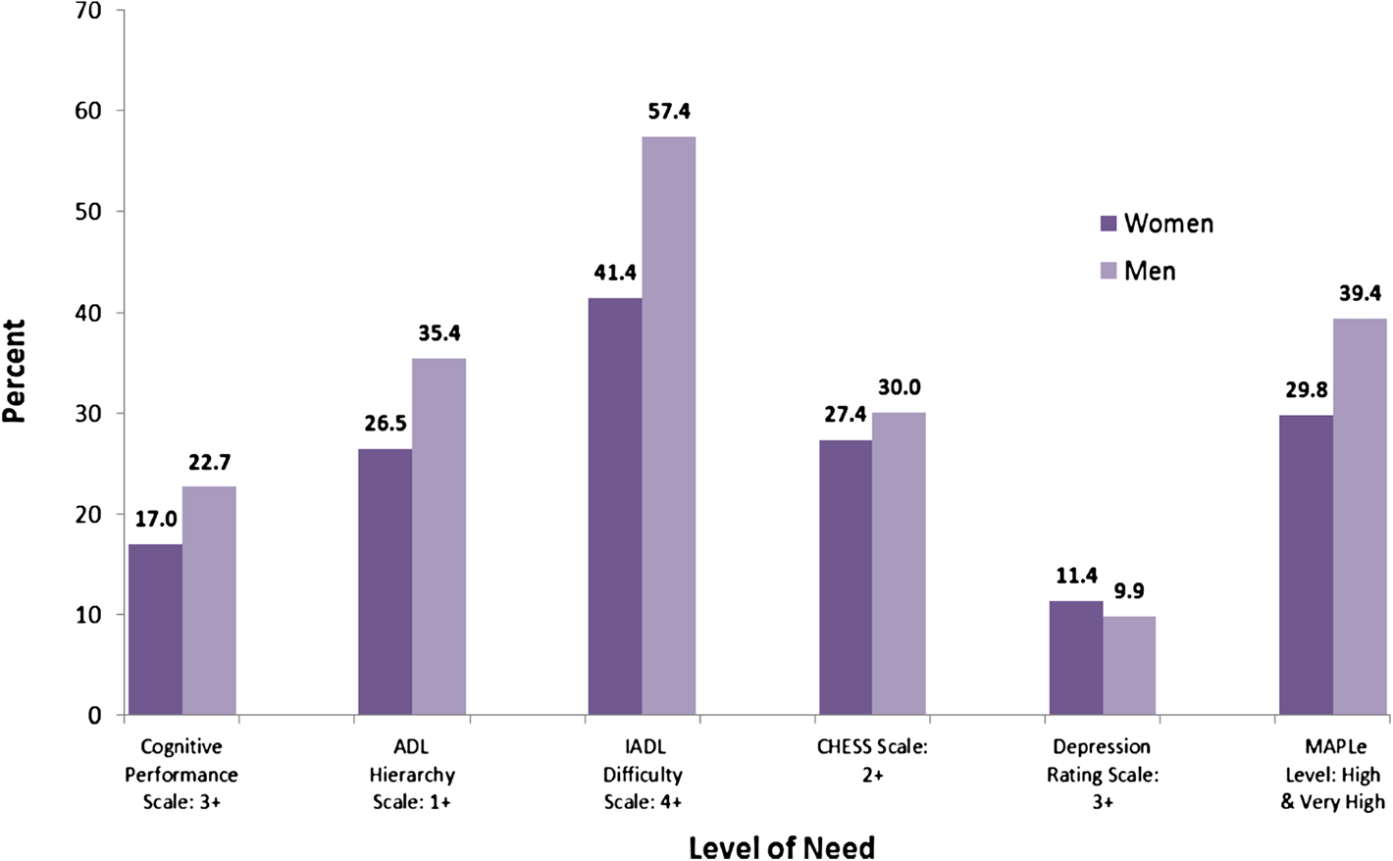
Proportion of older women and men in long-stay home care with cognitive and functional impairment, in Ontario, Canada, 2007.

Figure 2 displays the proportion of women and men who scored above the threshold on each of the RAI-HC outcome scales. Men were more likely to have at least moderate cognitive impairment (22.7% versus 17.0%) and limitations in their ADL (35.4% versus 26.5%) and IADL functioning (57.4% versus 41.4%). Men were also slightly more likely to be clinically unstable (30.0% versus 27.4%). Women, however, were somewhat more likely to exhibit depressive symptoms (11.4% versus 9.9%).

Men were more likely to visit the ED for any reason (59.2% versus 54.3%) and somewhat more likely to visit for potentially preventable conditions (20.7% versus 17.4%) than women; however, women were slightly more likely to visit the ED for fall-related injuries (12.8% versus 10.0%). Men were more frequently admitted to hospital than women (43.3% versus 34.5%). Of those admitted, men consistently showed longer lengths of stay (mean 25.5 days (standard deviation [SD] 37.2) versus 22.8 days (SD 33.2)) and were more likely to have received an ALC designation (14.5% versus 11.3%). Men were slightly more likely to apply for LTC (17.2% versus 15.4%) and to be admitted to LTC (12.5% versus 11.1%) during the follow-up year. Hospital use increased with age for women, but remained relatively constant for men (Table 2).

**Table 2:** Health services use during one year of follow-up among long-stay home care clients in Ontario, Canada, 2007 – overall and stratified by sex and age

	**Women N = 51,201**					**Men N = 20,102**				
	**All women**	**Aged 76-79**	**Aged 80-84**	**Aged 85-89**	**Aged 90+**	**All men**	**Aged 76-79**	**Aged 80-84**	**Aged 85-89**	**Aged 90+**
	**N = 51,201**	**N = 9,523**	**N = 16,447**	**N = 15,036**	**N = 10,195**	**N = 20,102**	**N = 4,781**	**N = 6,880**	**N = 5,413**	**N = 3,028**
**Acute care hospital use, % (95% CI)**										
**Emergency department visits**										
Any visits	54.3%	52.7%	53.2%	55.0%	56.7%	59.2%	57.3%	59.1%	60.3%	60.6%
	(53.7-55.0)	(51.2-54.1)	(52.1-54.3)	(53.8-56.2)	(55.2-58.2)	(58.2-60.3)	(55.2-59.5)	(57.3-60.9)	(58.3-62.4)	(57.9-63.5)
Visits for potentially prevenz conditions	17.4%	17.3%	17.2%	17.6%	17.6%	20.7%	19.2%	20.8%	21.0%	22.2%
	(17.0-17.8)	(16.4-18.1)	(16.6-17.9)	(16.9-18.3)	(16.8-18.4)	(20.1-21.3)	(18.0-20.5)	(19.8-21.9)	(19.8-22.2)	(20.6-23.9)
Visits for fall-related injuries	12.8%	10.5%	11.8%	13.5%	15.4%	10.0%	8.6%	9.7%	10.4%	12.4%
	(12.5-13.1)	(9.8-11.2)	(11.3-12.3)	(13.0-14.1)	(14.6-16.2)	(9.6-10.5)	(7.8-9.5)	(8.9-10.4)	(9.5-11.3)	(11.1-13.7)
**Acute care hospital admissions**										
Any admissions	34.5%	33.5%	33.9%	34.7%	36.4%	43.3%	42.3%	43.6%	43.5%	43.6%
	(34.0-35.1)	(32.3-34.6)	(33.0-34.8)	(33.7-35.6)	(35.2-37.6)	(42.4-44.2)	(40.5-44.2)	(42.0-45.1)	(41.8-45.3)	(41.3-46.0)
Average length of stay, mean ± SD	22.8 ± 33.2	22.9 ± 34.4	22.5 ± 34.4	23.2 ± 32.0	22.5 ± 32.2	25.5 ± 37.2	27.0 ± 41.2	26.0 ± 38.7	24.4 ± 33.9	24.3 ± 32.2
Any admissions with ALC	11.3%		10.5%	11.7%	13.6%	14.5%	12.2%	14.6%	15.1%	16.8%
	(11.0-11.6)	9.3% (8.7-	(10.0-11.0)	(11.2-12.3)	(12.9-14.3)	(14.0-15.0)	(11.2-13.2)	(13.7-15.5)	(14.1-16.2)	(15.4-18.3)
Average ALC length of stay for those with ALC days, mean ± SD	26.1 ± 40.8	26.1 ± 41.0	26.8 ± 45.6	26.0 ± 36.9	25.3 ± 39.2	29.3 ± 45.8	33.6 ± 55.4	29.4 ± 47.0	27.7 ± 40.5	26.8 ± 38.3
**Long-term care use, n (%)**										
Any long-term care applications	15.4%	11.8%	14.4%	16.4%	18.8%	17.2%	13.6%	16.6%	19.6%	19.7%
	(15.1-15.7)	(11.1-12.5)	(13.9-15.0)	(15.8-17.1)	(18.0-19.7)	(16.6-17.7)	(12.6-14.7)	(15.6-17.6)	(18.4-20.8)	(18.1-21.3)
Any long-term care placements	11.1%	7.8%	9.8%	12.2%	14.8%	12.5%	8.8%	12.4%	13.9%	15.9%
	(10.9-11.4)	(7.3-8.4)	(9.3-10.3)	(11.7-12.8)	(14.1-15.6)	(12.0-13.0)	(8.0-9.7)	(11.6-13.3)	(12.9-14.9)	(14.5-17.4)

## Discussion

In this study, we found important differences between the older women and men who use long-stay home care services in Ontario. These differences, evident across a range of factors including living situation and several health measures, are reflective of a different set of needs between older women and men that lead to home care use and subsequent health services outcomes. Although many of our findings are consistent with those on gender differences in home care entry and social support reported elsewhere [43-45], others are unique, in particular as they relate to overall impairment. Home care has become an integral component of the health care system and the key driver in reducing the more costly and less desirable alternatives of acute care hospital stays and LTC admissions. Ensuring that home care programs can meet that tall order requires a thorough understanding of the individuals who use these services and their outcomes. Our results and others reinforce the importance of considering gender in policy and planning for home care services.

As expected, we found that women receiving long-stay home care greatly outnumbered men overall and within each age group, and that women were disproportionately represented in the oldest age groups. This gender-split is even greater than that observed among all Ontarians in the same age brackets, suggesting that women accessed long-stay home care services more frequently than men [23]. Although we lack data on the reasons for seeking home care, we, along with others, did find significant differences in access to informal support as measured by living situation and presence of a spouse [5]. In every age group, including those over 90, women were more likely to live alone while men were more likely to live with their primary caregiver. For women, children or children-in-law were consistently identified as the most frequent primary caregiver whereas for men, spouses were most common except among the oldest age group. Men’s caregivers were twice as likely to report distress as women’s caregivers. Informal caregivers have often been described as the “backbone” of the health care system [43] and since home care generally does not provide round-the-clock care, successful programs rely on the involvement of such individuals. The different types of caregivers that older women and men rely on call for different types of caregiver support strategies. A spouse is likely also older and dealing with her own health concerns while an adult child is likely balancing work and childcare demands. An understanding of different caregiver needs, especially given other gendered differences among home care recipients and their caregivers, will be critical to building a stronger and more effective home care sector.

We also found that women and men differed in their clinical profiles. With few exceptions, men consistently had a higher prevalence of most chronic conditions. Men were also more likely to have greater impairment in IADL, ADL, and cognitive functioning as well as more clinical instability. On the other hand, women were more likely to have arthritis, osteoporosis and a history of fracture. Women and men experienced similarly high rates of multi-morbidity, and in both groups nearly 60% was prescribed 10 or more drugs. In some respects, these findings are inconsistent with those reported elsewhere. Others have generally reported a higher burden of multi-morbidity and disability among women, even within a home care population [1,2,23,44]. The differences between our cohort, other home care samples, and the general older population are likely a result of Ontario’s current home care referral and admission practices and policies. Relative to the broader population of older adults in Ontario, those receiving home care had a much greater burden of multimorbidity but without the gender differential more commonly observed. Further, home care recipients also exhibited a higher prevalence of nearly all chronic conditions than the general population, in particular dementia and a history of hip fracture, but with comparable gender differences [23]. Beyond these basic diagnostic measures, there is little population-based data that allows us to contrast the needs of home care recipients with the broader older population in Ontario or to evaluate the extent to which existing home care policies contribute to gender imbalances in health services use. A more thorough analysis of gender and home care access is beyond the scope of this study but is certainly necessary for future planning.

Although the prevalence of most chronic conditions was similar or higher among men than women, these findings need to be interpreted within the context of the substantial differences in the absolute numbers. The fact that women so greatly outnumbered men means that even where men were more likely to experience a condition, the number of women affected was higher. For instance, 37% of men had a diagnosis of COPD compared to 26% of women but in absolute terms there were nearly twice as many women affected. This does not diminish the significance of these conditions in men or their overall burden. It does however illustrate the importance of accounting for underlying population distributions in service planning, in particular for issues like gender which impact on the availability of supports outside the formal sector.

Lastly, we found that men were more likely than women to be admitted to hospital and to LTC during follow-up. Based on our previous work [23] and that of others [46-48], we had anticipated that a higher proportion of women relative to men would be admitted to LTC. We had also anticipated that women would have had longer hospital stays and more frequent ALC designations since these are both a function of LTC bed availability. Our findings on health services outcomes combined with gender differences in overall illness profile and caregiver support, including caregiver distress, suggest two possible and potentially related explanations. The first is that women access long-stay home care at earlier stages of disability; the second is that current service availability better meets the needs of women, which appear to be less medically intense. Future research should focus on gender differences in home care service type and length-of-stay, and disability at LTC admission. Such research could aid in the development of gender-specific strategies to improve home care quality and delay LTC admissions.

This study has limitations. First, we lack detailed data on degree of caregiver involvement and burden. We know that women receive fewer hours of informal care than men, [43] but it is less clear how this relates to the provision of formal services and health services outcomes. Second, as mentioned earlier, we were unable to provide detailed descriptions of the types of home care services received or the overall length-of-stay. Other research suggests that women are more likely to receive homemaking services while men are more likely to receive medical services [49]; given the gender profiles identified here, we would expect similar patterns but there is little information available to date. Lastly, this study includes data from Ontario only. Home care referral and admission policies vary across jurisdictions, both within Canada and internationally [50]. However, many jurisdictions have implemented the RAI-HC and collect administrative data comparable to that in Ontario. We believe that our study serves as model for gender analysis of home care users and highlights the importance of detailing the needs of each older women and men.

## Conclusions

Due to increasing emphasis on reducing hospital and LTC stays, home care has become an integral component in the care of older adults. Understanding who uses home care and why is critical to ensuring that these programs deliver the services that can best meet these objectives while enabling recipients to safely stay at home. We found that women significantly outnumbered men in home care but that men presented with higher levels of need and were more likely to be admitted to LTC. This detailed gender analysis highlights how needs differ between older women, men, and their respective caregivers. It will continue to be important to evaluate gender differences as home and community-based services evolve and as population demographics and gender roles continue to shift.

## Abbreviations

ADL: Activities of daily living; ALC: Alternate level of care; CCAC: Community care access centre; CHESS: Changes in health, end-stage disease, and signs and symptoms; CI: Confidence interval; COPD: Chronic obstructive pulmonary disease; CPS: Cognitive performance scale; DRS: Depression rating scale; ED: Emergency department; IADL: Instrumental activities of daily living; ICD: International classification of disease; LTC: Long-term care; MAPLe: Method for assigning priority levels; RAI-HC: Resident assessment instrument for home care; SD: Standard deviation.

## Competing interests

The authors have no competing interests to declare.

## Authors’ contributions

AG was involved in all aspects of this study including study conception and design, data analysis and interpretation, and drafting and critically revising the manuscript. JF was involved with study conception and assisted in drafting and critically revising the manuscript. XC was involved in study design, data analysis, and critically revising the manuscript. SG was involved in study conception, data interpretation, and revising the manuscript. SB was involved in study conception and design, data analysis and interpretation, critically revising the manuscript, and obtaining funding. All authors have approved the final version for publication.

## Pre-publication history

The pre-publication history for this paper can be accessed here:

http://www.biomedcentral.com/1471-2318/13/48/prepub

## Supplementary Material

Additional file 1 **Appendix A **– Diagnostic Definitions Using International Classification of Disease versions 9 and 10 Codes. Click here for file
