# Supplementary material for: Gender differences in home care clients and admission to long-term care in Ontario, Canada: a population-based retrospective cohort study
Source: BMC Geriatr. 2013 May 16;13:48. doi: 10.1186/1471-2318-13-48 (PMC3679828; doi:10.1186/1471-2318-13-48)
Supplement: Additional file 1 — Appendix A – Diagnostic Definitions Using International Classification of Disease versions 9 and 10 Codes. [file 1471-2318-13-48-S1.doc]

Appendix A

| *Diagnoses* | *ICD code* |
| --- | --- |
| Arthritis | ICD-10-CA: M05–M06 (rheumatoid arthritis); M15–M19 (osteoarthritis); M07, M10, M11–M14, M30–M36 (other inflammatory  and connective tissue diseases); M00–M03, M20–M25, M65–M79 (other arthritis and rheumatic conditions) or OHIP: 274 (gout); 446, 710 (connective tissue disorders); 711, 716, (traumatic arthritis, pyogenic arthritis); 714 (rheumatoid arthritis); 715 (osteoarthritis); 718, 728 (joint derangement, Dupuytren’s contracture); 720 (ankylosing spondylitis ); 727 (synovitis ); 729 (fibrositis); 739 (other MSK disorders) |
| COPD | ICD-10-CA: J10.0, J11.0, J12–J16, J18, J20, J21, J22, J41, J42, J43, J44, J47 Diagnosis type M (but not type M and 2) or ICD-10-CA: J10.0, J11.0, J12–J16, J18, J20, J21, J22, J41, J42, J43, J44, J47 Type 1, W, X, Y (but not also as type (2) with another diagnosis type M and 2) or OHIP: 491, 492, 496 |
| Cancer | ICD-10-CA: C00-C26, C30-C44, C45-C97, Z51.0, Z51.1 |
| Diabetes | In ICES Ontario Diabetes Database during each time period |
| Cardiovascular conditions | Acute myocardial infarction ICD-10-CA: I21.^, I22.^ Diagnosis type M (but not also as type 2) or ICD-10-CA: I21.^, I22.^ Type 1, W, X, Y (but not also as type 2 with another diagnosis type M and 2) or ICD-10-CA: Coronary artery disease I25.0, I25.1^, I25.8, I25.9 as type M, AMI as type 1, W, X, Y but not also as type 2, along with percutaneous coronary intervention 1.IJ.50^,  1.IJ.54.GQ–AZ, 1.IJ.57.GQ^ or coronary artery bypass graft 1.IJ.76.^  Congestive heart failure ICD-10-CA: I26.0, I27.9, I50.^ Diagnosis type M (but not also as type 2) or ICD-10-CA: I26.0, I27.9, I50.^ Type 1 (but not also as type 2 with another diagnosis type M and 2) or OHIP: 428  Stroke ICD-10-CA: I60.^, I61.^, I62.^, I63.^, I64 Diagnosis type M (but not also as type 2) or ICD-10-CA: I60.^, I61.^, I62.^, I63.^, I64 Type 1, W, X, Y (but not also as type 2 with another diagnosis type M and 2) |
| Urinary incontinence | ICD-10-CA: N393, N394, R32, R3914 |
| Dementia | Any 1 code/claim occurring during the 5-year look-back period ICD-10-CA (CIHI-DAD): F00.0, F00.1, F00.2, F00.9, F01.0, F01.1, F01.2, F01.3, F01.8, F01.9, F02.0, F02.1, F02.2, F02.3, F02.4, F02.8, F03.^, F05.1, F06.5, F06.6, F06.8, F06.9, F09.^, G30.0, G30.1, G30.8, G30.9, G31.0 G31.1, R54.^ or OHIP: 290, 331, 797 or Any cholinesterase inhibitor script in ODB during 1 year prior to index, ODB subclnam =: ‘CHOLINESTERASE INHIBITOR’ |
| Depression | Mental Health and Addictions ICD-10-CA codes (All Diagnosis Types): F320–323, F328–334, F338–339, F412, F480 or OMHRS records with a diagnosis in:  • Group 3 (“Major depression”) Q2AA, Q2AB, Q2AC, or Q2B = 296.20–296.39 or  • Group 5 (“Other unipolar depression”) Q2AA, Q2AB, Q2AC, or Q2B = 311^ |
| Other mental health conditions | ICD-10-CA codes (All Diagnosis Types): complete list available upon request or OMHRS records (from 2005/06 to 2008/09) with a Q2AA, Q2AB, Q2AC or Q2B diagnosis between DSM-IV 290^ to 347^, or in  (V6110, V6112, V6120, V6121, V6180, V6190, V6220, V6230, V6240, V6281, V6282) (where not in above dementia or depression code lists). |
| Fractures | ICD-10-CA: S22.0, S22.1 (thoracic spine); S32 (lumbar spine and pelvis); S42.2–S42.4, S42.7–S42.9 (shoulder/upper arm); S52, S62.0–S62.4, S62.8 (wrist/forearm); S72 (hip/femur); S82 (lower leg/ankle) |
| Osteoporosis | ICD-10-CA: M81, M82 or OHIP: 733 |
